# Enhanced Photocatalytic Activity of Z-Scheme Bi_2_WO_6_/P25 Heterojunctions via 7,7,8,8-Tetracyanoquinodimethane Modification

**DOI:** 10.3390/molecules31142472

**Published:** 2026-07-15

**Authors:** Yunxia Wei, Baolan Wang, Mingguang Ma, Fang Liu, Yichao Wang, Derek Hao

**Affiliations:** 1 College of Chemistry and Chemical Engineering, Lanzhou City University, Lanzhou 730070, China; 2 School of Science, RMIT University, Melbourne 3000, Australia

**Keywords:** heterojunction photocatalyst, heterojunction, TCNQ, charge separation

## Abstract

Efficient interfacial charge transfer is crucial for improving the photocatalytic performance of semiconductor heterojunctions under visible-light irradiation. In this study, Bi_2_WO_6_/P25 heterojunction photocatalysts modified with 7,7,8,8-tetracyanoquinodimethane (TCNQ) were prepared to enhance visible-light photocatalytic activity. The optimized sample with a TCNQ mass ratio of 0.3% exhibited the highest activity for rhodamine B degradation, achieving a degradation rate approximately 6.0 times higher than that of pure Bi_2_WO_6_ and 1.7 times higher than that of the pristine Bi_2_WO_6_/P25 heterojunction. The degradation rates of phenol were 6.5 times and 2.3 times higher for Bi_2_WO_6_ and BP-5, respectively. The enhanced photocatalytic performance was mainly attributed to the modification of TCNQ, which enhanced the electron transfer from P25 to Bi_2_WO_6_, establishing a multi-stage electron transfer mechanism involving P25 → Bi_2_WO_6_ → TCNQ. This molecular surface modification strategy provides new insights for the rational design of high-performance photocatalytic materials.

## 1. Introduction

Photocatalytic technology is an environmentally friendly green technology that degrades various organic pollutants through photocatalytic reactions under sunlight irradiation [1,2,3]. Compared with traditional biochemical degradation methods, photocatalytic technology offers advantages such as simple operation, environmental friendliness, broad applicability, and mild reaction conditions, making it highly promising for environmental remediation [4]. However, most photocatalysts suffer from limited light absorption and rapid recombination of photogenerated electron-hole pairs, resulting in unsatisfactory photocatalytic activity [5]. For instance, Bi_2_WO_6_, with a narrow bandgap of 2.70 eV, demonstrated visible-light absorption response, excellent stability, and low toxicity, and has therefore attracted considerable attention [6,7,8]. Nevertheless, the rapid recombination of photo-induced electron-hole pairs and low quantum efficiency still restrict its photocatalytic performance. To address these challenges, coupling Bi_2_WO_6_ with suitable semiconductors to form heterojunctions can facilitate interfacial charge separation and broaden the light-response range, thereby improving photocatalytic efficiency [9,10,11]. Previous studies have employed one-step solvothermal methods to prepare Z-scheme Bi_2_WO_6_/P25 heterojunctions [12,13]. Compared with P25, the absorption edge of Bi_2_WO_6_/P25 shifts significantly toward longer wavelengths (red shift); however, this redshift is less pronounced than that observed in Bi_2_WO_6_ alone [13].

7,7,8,8-Tetracyanoquinodimethane (TCNQ) is a strong organic electron acceptor, composed of a quinodimethane-type conjugated framework bearing four strongly electron-withdrawing cyano groups. The conjugated system, combined with four strongly electron-withdrawing cyano groups, is endowed with an extremely low LUMO energy level (4.6 eV), making it highly prone to electron donation [14]. When in contact with a semiconductor, it can efficiently extract and store photogenerated electrons from the semiconductor [15]. When interacting with semiconductors, TCNQ participates in forming charge transfer channels, significantly enhancing the photocatalyst’s light-responsive capability and overall performance across a broader spectral range [16]. Therefore, modifying the Bi_2_WO_6_/P25 heterojunction with TCNQ is expected to enhance photocatalytic performance by facilitating interfacial electron transfer.

Inspired by this electron-mediation concept, TCNQ-modified Bi_2_WO_6_/P25 heterojunction photocatalysts (BP-5@TCNQ) were constructed via a facile post-modification method involving solution impregnation and solvent evaporation. As a strong electron acceptor, TCNQ effectively captures electrons from the Bi_2_WO_6_/P25 heterojunction, thereby establishing a directional electron transport pathway from the catalyst bulk to TCNQ. This structure not only enhances the separation efficiency of photogenerated carriers but also effectively suppresses electron-hole pair recombination.

## 2. Results and Discussion

### 2.1. Screening of Catalysts

**Figure 1:**
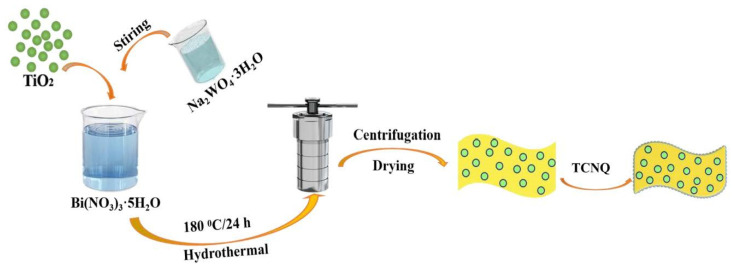
Schematic illustration of the preparation of the BP@TCNQ composite photocatalyst.

**Figure 2:**
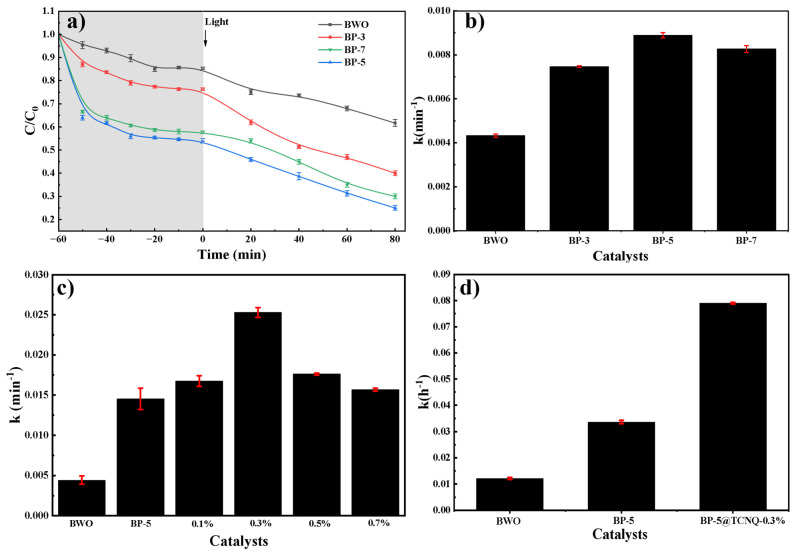
Photocatalytic activities of BP composite materials: (**a**) photocatalytic degradation curve of BP composite photocatalysts for the degradation of 10 ppm RhB with different P25 mass under visible light (λ > 420 nm); (**b**) apparent rate constants k of BP composite photocatalysts for the degradation of RhB; (**c**) apparent rate constants k of Bi_2_WO6, BP-5, and BP-5@TCNQ composite photocatalysts for the degradation of RhB; (**d**) apparent rate constants k of Bi_2_WO_6_, BP-5, and BP-5@TCNQ-0.3% photocatalysts for the degradation of phenol.

Figure 1 shows the flowchart for the preparation of the photocatalyst. As shown in Figure 2a, BP-5 delivered the best photocatalytic performance among pure Bi_2_WO_6_ and the BP-series composites. In addition, Figure 2b shows that the BP composites exhibited stronger adsorption toward RhB than pure Bi_2_WO_6_, with BP-5 showing the highest adsorption capacity. This behavior is consistent with its larger specific surface area (Table 1), which provides more accessible adsorption and reaction sites. Nevertheless, adsorption alone is unlikely to fully account for the enhanced photocatalytic activity of the BP composites. The improved activity is more likely associated with the formation of a Bi_2_WO_6_/P25 heterojunction, which can facilitate charge separation and interfacial electron transfer. Figure 2c demonstrates the photocatalytic performance of BP-5@TCNQ-x% in degrading RhB under visible light (λ > 420 nm). The catalytic activity of the series of photocatalysts first improved and then declined with increasing TCNQ content. Notably, when the TCNQ content reached 0.3%, the catalyst (BP-5@TCNQ-0.3%) exhibited optimal performance (k = 0.0249 min^−1^), nearly 1.7 times that of BP-5 (0.0155 min^−1^) and 6.0 times that of Bi_2_WO_6_ (0.0043 min^−1^). Figure 2d presents the photocatalytic activities of the photocatalysts under visible light (λ > 420 nm) in the degradation of phenol. Compared with Bi_2_WO_6_ and BP-5, under identical conditions, BP-5@TCNQ-0.3% exhibited the highest degradation rate constant for 5 ppm phenol at 0.0790 h^−1^, which is 6.5 times and 2.3 times higher than those of Bi_2_WO_6_ and BP-5, respectively.

The processes of phenol degradation and intermediate formation were investigated by high-performance liquid chromatography (HPLC) (Figure S2). The peak at 3.557 min represents phenol; the intensity decreased after 4 h of irradiation as a result of the photocatalytic reaction. No background peaks are observed in the figure, indicating that phenol has been completely mineralized.

### 2.2. Structure of the Catalysts

#### 2.2.1. Morphology and Structure Analysis

**Figure 3:**
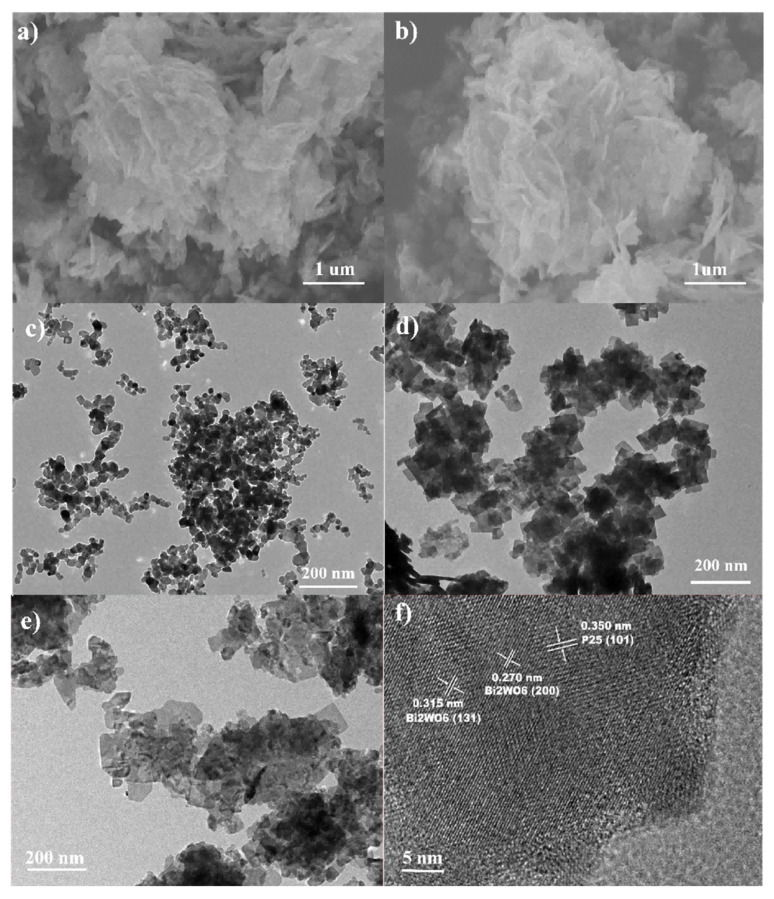
SEM images of Bi_2_WO_6_ (**a**), BP-5 (**b**); TEM image of P25 (**c**), Bi_2_WO_6_ (**d**) and BP-5 (**e**); HRTEM image of magnified BP-5 (**f**).

The morphology of the material was observed using SEM, and the microstructure of the material was analyzed using TEM. The results are shown in Figure 3. Figure 3a,b show that Bi_2_WO_6_ and BP-5 were both flower-like. As shown in Figure 3c,d, P25 and Bi_2_WO_6_ were nanoparticle and nanosheet structures, respectively. The results demonstrate that Bi_2_WO_6_ prepared by this method exhibited a nanosheet structure, which is consistent with previous reports [17,18,19]. Figure 3e shows that the P25 nanoparticles were uniformly distributed in five nanosheets, which was significantly lower than that in Bi_2_WO_6_.

Furthermore, as shown in Figure 3f, the lattice fringes of 0.270 and 0.315 nm corresponded to the (200) and (131) planes of Bi_2_WO_6_, respectively. The lattice fringes of 0.350 nm in the HRTEM image corresponded to the (101) plane of P25.

Based on the above characterization results, it can be concluded that a heterojunction was formed between Bi_2_WO_6_ and P25.

#### 2.2.2. The Interaction Between TCNQ and BP-5

**Figure 4:**
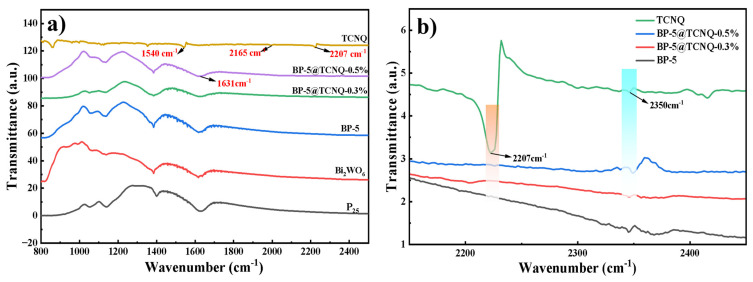
FTIR patterns of Bi_2_WO_6_, P25, TCNQ, BP-5, BP-5@TCNQ-0.3%, and BP-5@TCNQ-0.3% (**a**) and its enlarged drawing (**b**).

The infrared spectrum (Figure 4) shows that pure TCNQ exhibits characteristic absorption peaks at 2207 cm^−1^, 2165 cm^−1^, and 1540 cm^−1^, corresponding to -C≡N stretching vibration and benzene ring C=C stretching vibration, respectively [20].

When TCNQ was modified onto the surface of the Bi_2_WO_6_/P25 heterojunction, the W-O bond vibration peak of Bi_2_WO_6_ in BP-5@TCNQ-x% widened and shifted, while the intensity of the -C≡N characteristic peak of TCNQ decreased and widened, indicating that Bi^3+^ on the surface of Bi_2_WO_6_ formed a coordination bond with the -C≡N of TCNQ, and there was a strong interaction between the two [21].

For P25, the O-H bending vibration peak of the adsorbed water on its surface shifted from 1630 cm^−1^ to 1631 cm^−1^, and its intensity increased. Simultaneously, the Ti-O bond vibration peak widened, indicating that TCNQ formed hydrogen bonding with the Ti-OH on the P25 surface. In addition, the C=C vibration peak of TCNQ’s benzene ring underwent a red shift, indicating the existence of a charge transfer interaction between the two [22].

These results indicate that TCNQ interacts with Bi_2_WO_6_ and P25 simultaneously, constructing a more efficient interface charge transfer channel, which provides an important structural basis for the improvement of heterojunction photocatalytic performance.

#### 2.2.3. XPS

**Figure 5:**
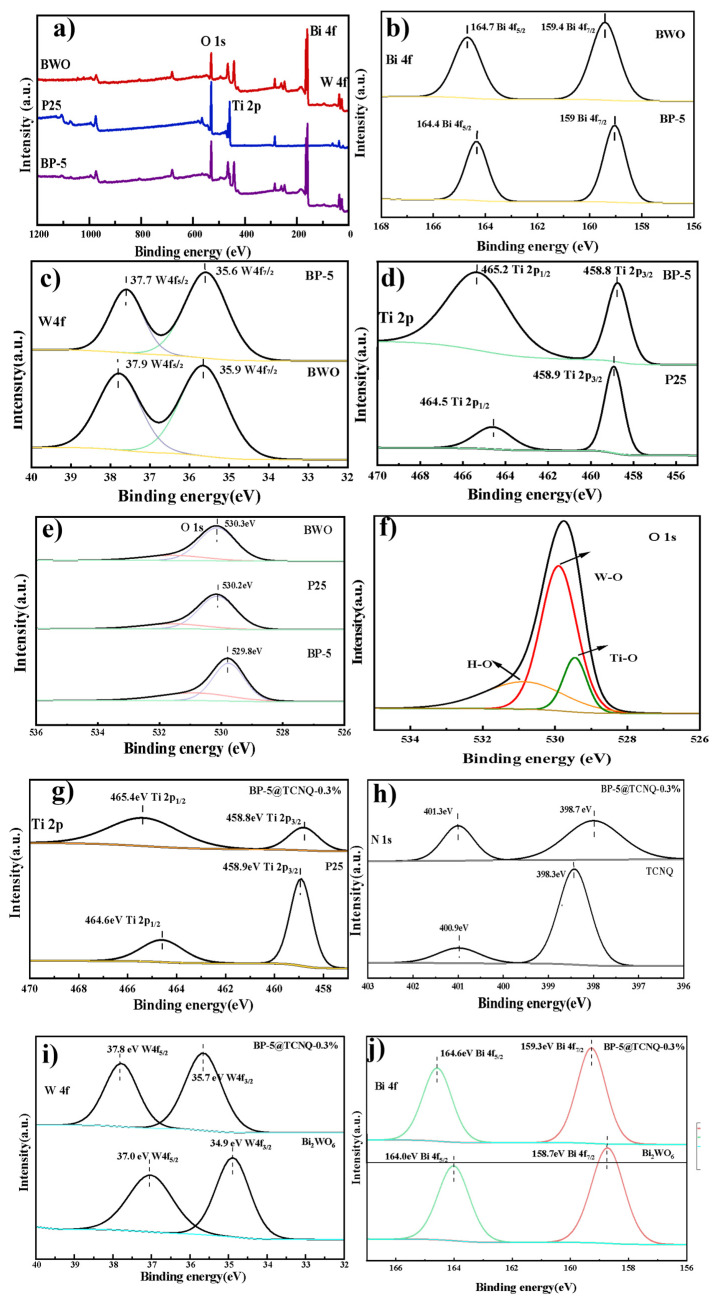
XPS spectra of Bi_2_WO_6_, P25, BP-5, and BP-5@TCNQ-0.3%: (**a**) survey; (**b**) Bi 4f; (**c**) W 4 f; (**d**) Ti 2p; (**e**,**f**) O 1s; (**g**) Ti 2p in P25 and BP-5@TCNQ-0.3%; (**h**) N 1sp in TCNQ and BP-5@TCNQ-0.3%; (**i**) W 4f in Bi_2_WO_6_ and BP-5@TCNQ-0.3%; (**j**) Bi 4f in Bi_2_WO_6_ and BP-5@TCNQ-0.3%.

XPS full spectrum analysis (Figure 5a) showed that the characteristic peaks of Bi, W, Ti, and O appeared simultaneously in BP-5, which, combined with the HRTEM (Figure 3f) results, once again confirmed the successful recombination of Bi_2_WO_6_ and P25 to form a heterojunction [23].

As shown in Figure 5b, the binding energies of Bi 4f _5_/_2_ and Bi 4f _7_/_2_in Bi_2_WO_6_ are 164.7 eV and 159.4 eV, respectively, whereas in BP-5, these values shift negatively to 164.4 eV and 159.0 eV. The reduced binding energy indicates an increased electron cloud density around the Bi atoms in BP-5, demonstrating that electrons have been transferred from P25 to Bi_2_WO_6_.

The binding energies of W 4f _5_/_2_ and W 4f _7_/_2_ in Bi_2_WO_6_ are 37.9 eV and 35.9 eV, respectively; in BP-5, these energies shift negatively to 37.7 eV and 35.6 eV (Figure 5c), indicating an increase in electron cloud density around the W atoms and further confirming that electrons have been transferred from P25 to Bi_2_WO_6_.

Simultaneously, the binding energy of the Ti 2p orbital in PB-5 exhibits a positive shift (Figure 5d), further confirming the directional migration of electrons from P25 to Bi_2_WO_6_. This interface charge redistribution establishes an intrinsic electric field that effectively suppresses the recombination of photogenerated carriers.

As shown in Figure 5e, the binding energy of O 1s in Bi_2_WO_6_ is 530.3 eV, while that in P25 is 530.2 eV. In BP-5, the binding energy of O 1s shifts negatively to 529.8 eV; this decrease indicates an increased electron cloud density around the oxygen atoms, suggesting their participation in the electron transfer process at the interface [24].

In Figure 5g, the binding energies of Ti 2p _1/2_ and Ti 2p _3/2_ in BP-5@TCNQ-0.3% shift positively to 465.4 eV and 458.8 eV, respectively. Compared with BP-5 (Figure 5d), the binding energy of Ti 2p increases further, indicating that P25 loses more electrons and the electron transfer is enhanced [25,26].

The binding energies of N 1s in TCNQ are 398.3 eV (cyano-N) and 400.9 eV (defect-state N), respectively; in BP-5@TCNQ-0.3%, these values shift upward to 398.7 eV and 401.3 eV, indicating a 0.4 eV increase (Figure 5h), which reflects reduced electron cloud density around the N atoms and demonstrates that TCNQ has accepted electrons from BP-5.

The binding energies of pure BWO’s W 4f_5/2_ and W 4f_7/2_ are 37.0 eV and 34.9 eV, respectively; in BP-5@TCNQ-0.3%, they increase to 37.8 eV and 35.7 eV, indicating electron loss from the W atoms (Figure 5i). The binding energies of pure BWO’s Bi 4f_5/2_ and Bi 4f_7/2_ are 164.0 eV and 158.7 eV, respectively; in BP-5@TCNQ-0.3%, they shift to 164.6 eV and 159.3 eV, indicating an increase in binding energy due to electron loss from the Bi atoms (Figure 5j).

Based on the above analysis, after modification with TCNQ, electrons transfer from the Bi_2_WO_6_/P25 heterojunction to TCNQ, which acts as an electron acceptor and further facilitates the separation of photogenerated charges.

In summary, after the heterojunction BP-5 is modified with TCNQ, the electron transfer pathway among P25, Bi_2_WO_6_, and TCNQ is: P25 → Bi_2_WO_6_ → TCNQ. This multi-stage electron transfer mechanism effectively enhances the separation of photogenerated carriers, which is a key factor contributing to the superior photocatalytic performance of BP-5@TCNQ-0.3%.

#### 2.2.4. XRD

**Figure 6:**
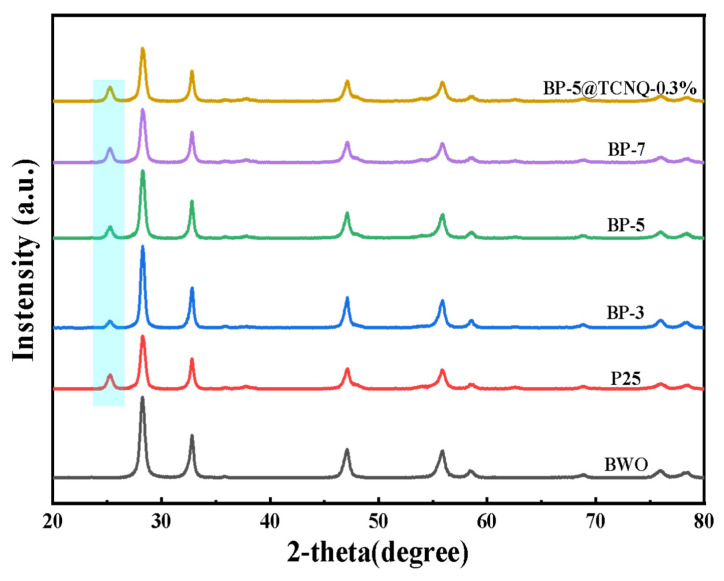
XRD patterns of Bi_2_WO_6_, P25, BP-3, BP-5, BP-7, and BP-5@TCNQ-0.3%.

The XRD patterns of the series of photocatalysts are shown in Figure 6. Compared with those of Bi_2_WO_6_ and P25, the XRD patterns of the BP series (BP-3, BP-5, and BP-7) exhibit characteristic peaks of both Bi_2_WO_6_ and P25 without additional interference peaks, indicating that the composite materials retained their respective crystalline phases [27]. The BP-x peak at 2θ = 25.2° demonstrated increasing intensity with higher P25 content, confirming the uniform surface dispersion of P25 on Bi_2_WO_6_ and the absence of localized agglomeration. This demonstrates the formation of homogeneous heterojunction interfaces that maximize the efficiency of photogenerated carrier separation. Moreover, the modification with TCNQ did not alter the crystal structure of the original heterojunction.

### 2.3. Photocatalytic Degradation Mechanism

**Figure 7:**
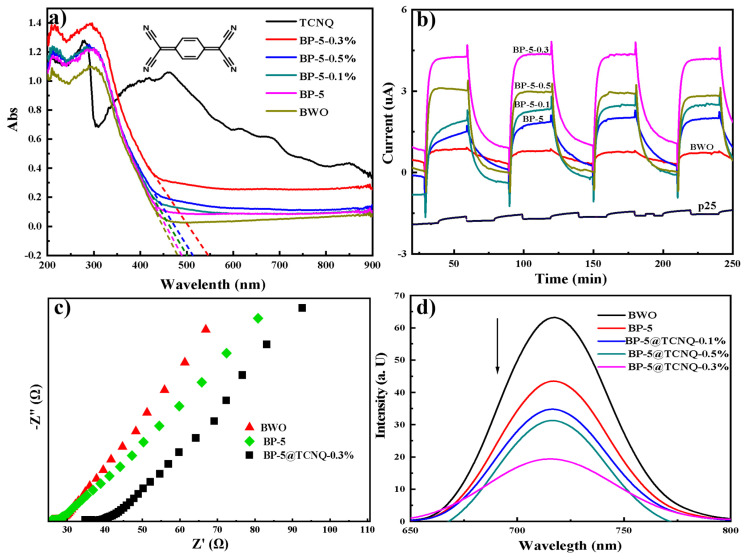
UV-DRS spectra of Bi_2_WO_6_, BP-5, and BP-5@TCNQ, the (**a**) shows the structural diagram of TCNQ; (**b**) photocurrent spectra; (**c**) EIS spectra; (**d**) fluorescence spectra.

In the UV-DRS spectrum (Figure 7a), Bi_2_WO_6_ exhibits only strong absorption in the ultraviolet region. The absorption of BP-5 in the visible-light region was slightly enhanced by the introduction of P25 to construct the Bi_2_WO_6_/P25 (BP-5) heterojunction. When the heterojunction was modified with TCNQ (BP-5@TCNQ-x%), the absorption intensity of visible light was significantly improved, especially when the TCNQ content was 0.3% (BP-5@TCNQ-0.3%). The absorption edge of the series of catalysts gradually redshifted with an increase in the TCNQ content, indicating the formation of charge transfer complexes between TCNQ and the heterojunctions, which effectively expanded the photoresponsive range. The conjugated structure of TCNQ can serve as an electron acceptor, promoting the separation of photogenerated charge carriers. Simultaneously, its broad spectral absorption characteristics further enhance the light-capture ability of the sample, providing favorable conditions for visible-light-driven photocatalytic reactions.

The separation efficiency of photogenerated electrons and holes at the interface of photocatalysts is a key factor in evaluating their photocatalytic activity. The charge separation efficiency of photogenerated electrons and holes at the material interface of a series of photocatalysts was studied by measuring the photocurrent of the electrodes, as shown in Figure 7b. The instantaneous photocurrent response spectrum (Figure 7b) shows that the series of photocatalysts exhibit better reversible photoresponsiveness in the light switch cycle, proving that the series of photocatalysts have excellent photostability. The photocurrent signal of P25 is extremely weak, while that of Bi_2_WO_6_ exhibits a clear positive photocurrent; when Bi_2_WO_6_ and P25 form a heterojunction, the photocurrent of BP-5 significantly increases, indicating that the heterojunction structure effectively promotes the separation of photogenerated carriers in the material. After being modified by TCNQ, the photocurrent of the series of photocatalysts was further enhanced, with BP-5-0.3% having the highest photocurrent value. An appropriate amount of TCNQ as an electron acceptor can quickly capture photogenerated electrons in heterojunctions and suppress carrier recombination; when the content of TCNQ is increased to 0.5%, the photocurrent of the material decreases, which may be due to the excessive aggregation of TCNQ and the formation of carrier recombination centers.

The heterojunction photocatalyst modified with TCNQ (BP-5@TCNQ-0.3%) exhibited significantly reduced interfacial charge transfer resistance (Figure 7c), indicating that TCNQ modification lowers the electron transfer barrier within the catalyst [28]. Combined with photocurrent test results (Figure 7b), it is evident that the fluorescence intensity of the TCNQ-modified heterojunction photocatalyst (BP-5) decreased (Figure 7d), while the photocurrent density increased (Figure 7b). XPS data further elucidate this mechanism: as a strong electron acceptor, TCNQ effectively captures electrons from the Bi_2_WO_6_/P25 heterojunction, establishing a directional electron transport pathway from the catalyst bulk to TCNQ. This configuration not only enhances the separation of photogenerated carriers but also effectively suppresses electron-hole pair recombination, thereby explaining the intrinsic improvement in the photocatalytic performance of BP-5@TCNQ-0.3%.

To identify the primary active species involved in the photocatalytic degradation of RhB by BP-5@TCNQ-0.3%, we conducted free radical trapping experiments using EDTA-2Na (hole h^+^ trap), p-BQ (superoxide radical O_2_^−^ trap), and K_2_Cr_2_O_7_ (electron e^−^ trap), with the system without any trap added serving as a control. Figure S3 shows that after addition of EDTA-2Na (hole trap), the degradation rate of RhB was only approximately 10%, indicating that holes (h^+^) are the dominant active species in the degradation process. With p-BQ (superoxide radical trap), the degradation rate reached about 15%, suggesting participation of O_2_^−^ but with a relatively minor contribution. Addition of K_2_Cr_2_O_7_ (electron trap) still achieved a degradation rate of around 60%, demonstrating that photogenerated electrons (e^−^) contribute minimally directly to degradation, primarily acting indirectly through reaction with O_2_ to form O_2_^−^. The C/C_0_ ratio remained nearly constant at 1, indicating negligible degradation of RhB and ruling out self-decomposition as the mechanism.

**Figure 8:**
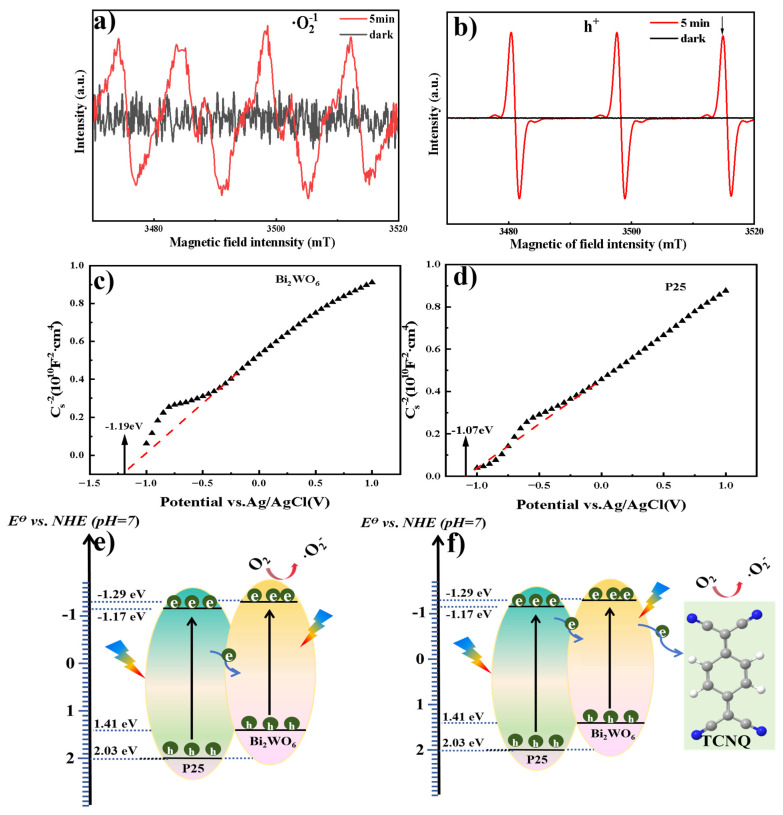
EPR spectra of BP-5@TCNQ-0.3% (**a**) in methanol for DMPO-O_2_^−^ and (**b**) in aqueous solution for TEMPO-h^+^; (**c**) Mott–Schottky curves for Bi_2_WO_6_ and (**d**) P25; (**e**) schematic diagram of charge transfer in BP-5; (**f**) schematic diagram of charge transfer in BP-5@TCNQ-0.3%.

The in situ active species during the catalytic process were further detected by electron paramagnetic resonance (ESR); the results are shown in Figure 8a,b. The results showed that ·O_2_^−^ and h^+^ involved in the photocatalytic oxidation reaction were effectively detected after illumination. In summary, O_2_^−^ and h^+^ are the main active species for the photocatalytic degradation of RhB, and they dominate the photocatalytic oxidation process.

The flat band potential of Bi_2_WO_6_ was measured via the Mott–Schottky curve (Figure 8c) to be −1.19 eV relative to NHE; since the conduction band of n-type semiconductors is typically 0.1–0.2 eV below this flat band potential, the conduction band minimum of Bi_2_WO_6_ is thus determined as −1.29 eV relative to NHE [29]. Combining this with the reported bandgap width of Bi_2_WO_6_ (2.7 eV), the valence band maximum was calculated using the formula E_VB_ = E_CB_ + Eg to be 1.41 eV relative to NHE. Similarly, for P25, the flat band potential is −1.07 eV relative to NHE, and the conduction band minimum is −1.17 eV relative to NHE (Figure 8d); combining this with the reported bandgap width of P25 (3.2 eV), the valence band maximum was calculated to be 2.03 eV relative to NHE.

Based on the above results, the mechanism of the photocatalytic reaction is depicted in Figure 8e,f. When the heterojunction BP-5 is modified with TCNQ, the electron transfer pathway among P25, Bi_2_WO_6_, and TCNQ is P25 → Bi_2_WO_6_ → TCNQ. This multi-stage electron transfer mechanism effectively enhances the separation of photogenerated carriers, which is a key factor contributing to the superior photocatalytic performance of BP-5@TCNQ-0.3%.

### 2.4. The Reusability and Stability of Photocatalysts

**Figure 9:**
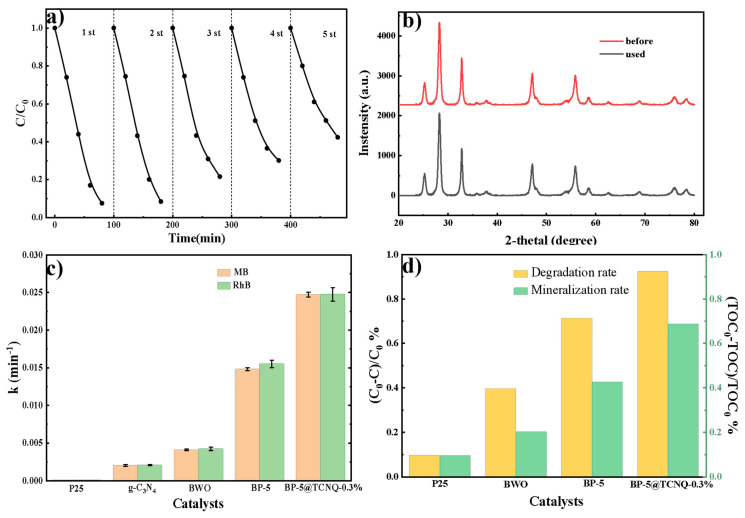
(**a**) Stability of BP-5@TCNQ-0.3% photocatalytic degradation of RhB; (**b**) XRD spectra of BP-5@TCNQ-0.3% photocatalyst before and after five cycles of RhB degradation experiments; (**c**) comparison of degradation performance of RhB and MB by several common photocatalysts under the same conditions; (**d**) comparison of degradation and mineralization rates of RhB by P25, Bi_2_WO_6_, BP-5, and BP-5@TCNQ-0.3%.

The stability of the BP-5@TCNQ-0.3% composite was evaluated through five cycling experiments. As shown in Figure 9a, during the first cycle, the catalyst achieved a degradation rate of 90% for RhB within 80 min, corresponding to the highest first-order reaction rate constant k, indicating excellent initial catalytic activity. After two cycles, the degradation rate remained at 80%, demonstrating good cycling stability of this heterojunction photocatalyst. However, the catalytic activity significantly declined by the third cycle: firstly, partial recovery of the photocatalyst was unavoidable due to its nanoscale size and poor dispersibility in water; secondly, repeated cycling led to adsorption of organic pollutant intermediates on the catalyst surface, which blocked active sites and reduced carrier separation efficiency. Moreover, as shown in Figure 9b, there was no obvious difference in the XRD of BP-5@TCNQ-0.3% before and after the reaction, indicating that the crystal structure of the catalyst exhibited excellent stability. According to the above analysis, it was concluded that the BP-5@TCNQ-0.3% composite was a stable photocatalyst with great application potential for wastewater treatment. Furthermore (Figure 9c), compared with several commonly used catalysts such as g-C_3_N_4_ nanosheets and Bi_2_WO_6_, the BP-5@TCNQ-0.3% heterojunction also has better photocatalytic activity in degrading other dyes (such as methylene blue). The mineralization of RhB in the photocatalytic reaction was analyzed by the TOC measurement. As can be seen from Figure 9d, when the irradiation time was 80 min, the degradation rate and TOC removal efficiency of RhB by BP-5@TCNQ-0.3% were 92.5% and 70.3%, respectively, which were better than those of Bi_2_WO_6_ and BP-5. This indicates that after modification with TCNQ, the degradation ability of the catalyst has been improved, while mineralization ability has also been enhanced.

## 3. Experimental Section

### 3.1. Materials

Bismuth nitrate (Bi(NO_3_)_3_·5H_2_O) and sodium tungstate (Na_2_WO_4_·2H_2_O) were purchased from Heowns Biochemical Technology Co., Ltd. (Tianjin, China). TCNQ was purchased from Beijing Chemical Reagent Corp., Beijing, China. All chemicals were of analytical grade and used without further purification.

### 3.2. Catalyst Preparation

Bi_2_WO_6_ was prepared according to a previously described method [30]. The Bi_2_WO_6_/P25 composites (BP) were prepared using a one-step hydrothermal method [12]. It should be noted that the materials were named BP-X (X = 3, 5, 7) based on the varying content of P25 (0.3, 0.5, 0.7 g) in the Bi_2_WO_6_/P25 composite.

The BP-5@TCNQ photocatalysts were prepared as follows: First, a tetrahydrofuran (THF) solution of TCNQ with a concentration of 5 g·L^−1^ was prepared. Different volumes of TCNQ solution were added to a container containing 0.3 g of BP-5 and diluted with ethanol to a volume of 25 mL. The mixture was vigorously stirred in a fume hood until the THF completely evaporated, and the solid was dried at 80 °C in a vacuum oven for subsequent use. The produced BP-5@TCNQ photocatalysts with various proportions of TCNQ were labeled BP-5@TCNQ-X% (X = 0.1, 0.3, 0.5, and 0.7).

### 3.3. Characterizations

The crystal structures of the as-prepared samples were examined by X-ray diffraction (XRD) on a Bruker D8 ADVANCE diffractometer using Cu Kα radiation (Rigaku Ulitma IV, Akishima, Tokyo). The morphology and microstructure were investigated by high-resolution transmission electron microscopy (HRTEM, JEM-2010F, Kumamoto, Japan) operated at an accelerating voltage of 200 kV. Fourier-transform infrared (FTIR) spectra were obtained using a Bruker V70 FTIR spectrometer (Bruker tensor 27, Bremen, Germany). UV–vis diffuse reflectance spectra (UV–vis DRS) were recorded on a UV–vis diffuse reflection spectrum using BaSO_4_ as the reference (Hitachi U-3010 UV, Tokyo, Japan). Photocurrent responses were measured with a electrochemical workstation (CHI-660B, Austin, TX, USA). N_2_ adsorption–desorption isotherms were obtained using a surface area and porosity analyzer (ASAP2460, Norcross, GA, USA). Total Organic Carbon (TOC) was determined using the combustion oxidation-non-dispersive infrared absorption method (TOC-5000A, Shimadzu, Kyoto, Japan).

### 3.4. Photocatalytic Experimental Procedure

The photocatalytic activities of the as-prepared samples were evaluated by degrading rhodamine B (RhB) (10 mg L^−1^) and phenol (5 mg L^−1^) respectively under visible-light irradiation. In a typical experiment, 25 mg of photocatalyst was added to 50 mL of RhB aqueous solution (10 mg L^−1^) or phenol aqueous solution (5 mg L^−1^) in a quartz tube. Prior to irradiation, the suspension was stirred in the dark for 1 h to reach adsorption–desorption equilibrium. The reaction was performed in a PCX50C multi-channel photochemical reactor (Pofei Company, Beijing, China) equipped with a white LED light source (λ > 420 nm). The LED current, stirring speed, and lamp panel switching interval were set to 60%, 350 rpm, and 20 s, respectively. The reaction was conducted at room temperature. At predetermined intervals, 2 mL aliquots were withdrawn, and the residual concentration of RhB was measured using a UV–vis spectrophotometer. The determination of phenol concentration was performed using a high-performance liquid chromatograph (HPLC) with a Venusil XBP-C18 column at a detection wavelength of 270 nm for phenol.

## 4. Conclusions

TCNQ-modified Bi_2_WO_6_/P25 heterojunction photocatalysts (BP@TCNQ) were prepared by a simple method. TCNQ forms chemical bonds with both P25 and Bi_2_WO_6_, respectively, in BP. The modification of TCNQ broadens the absorption range of the heterojunction photocatalyst (BP) for visible light, enhances the current density of the heterojunction catalyst, reduces the interfacial charge transport resistance, and enhances the electron transfer from P25 to Bi_2_WO_6_. When the heterojunction BP-5 is modified with TCNQ, the electron transfer pathway among P25, Bi_2_WO_6_, and TCNQ is P25 → Bi_2_WO_6_ → TCNQ. This multi-stage electron transfer mechanism effectively promotes the separation of photogenerated carriers, which is a key reason for the superior photocatalytic performance exhibited by BP-5@TCNQ-0.3%.

## Figures and Tables

**Table 1:** BET surface area, average pore diameter, and pore volume of all catalysts.

Catalysts	BET Surface Area (m^2^·g^−1^)	Average Pore Diameter (Å)	Pore Volume (cm^3^·g^−1^)
Bi_2_WO_6_	23.9	53.13	0.0332
P25	92.6	63.92	0.1661
BP-3	69.9	58.18	0.0631
BP-5	80.2	61.71	0.1371
BP-7	78.1	60.12	0.0511

## Data Availability

The data supporting the findings of this study are available from the corresponding author upon reasonable request.

## Supplementary Materials

The following supporting information can be downloaded at: https://www.mdpi.com/article/10.3390/molecules31142472/s1, Figure S1: Adsorption experiment of Bi_2_WO_6_ and the heterojunction photocatalyst BP-x (RhB concentration: 10 mg/L; temperature: 25 °C); Figure S2: HPLC map of initial 5 ppm phenol to the map of phenol after 4 h of photocatalytic degradation; Figure S3: Effect of catalysts on the degradation of RhB after adding different scavengers [Reaction conditions: RhB = 10 mg·L^−1^ (50 mL), LED light source (λ > 420 nm), BP-5@TCNQ-0.3% dosage = 0.5 g·L^−1^, EDTA-2Na = 10 mM, p-BQ = 1 mM, K_2_Cr_2_O_7_ = 0.5 mM, reaction time = 80 min, reaction temperature = 25 °C].
