# Supplementary material for: Enhanced Photocatalytic Activity of Z-Scheme Bi2WO6/P25 Heterojunctions via 7,7,8,8-Tetracyanoquinodimethane Modification
Source: Molecules. 2026 Jul 15;31(14):2472. doi: 10.3390/molecules31142472 (PMC13415069; doi:10.3390/molecules31142472)
Supplement: Supplementary file 1 [file molecules-31-02472-s001.zip › molecules-4398416-supplementary.pdf]

Enhanced Photocatalytic Activity of Z-Scheme Bi_2WO_6/P25 Heterojunctions via 7,7,8,8-Tetracyanoquinodimethane Modification

1. Catalyst Adsorption Experiment

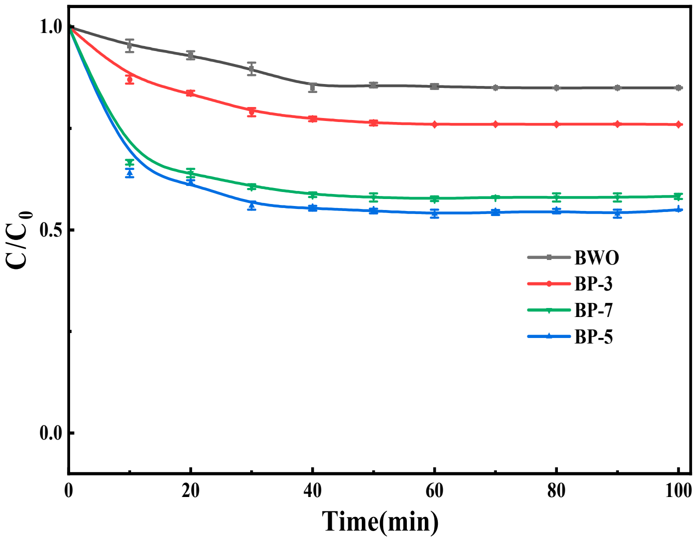

Figure S1. Adsorption experiment of Bi_2WO_6 and the heterojunction photocatalyst BP-x (RhB concentration: 10 mg/L; temperature: 25°C)

In photocatalytic degradation reactions, pollutants must first adsorb onto the catalyst surface before subsequent photocatalytic degradation can occur. Therefore, the adsorption capacities of various catalysts for target pollutants were investigated. The results demonstrated that all catalysts reached adsorption–desorption equilibrium after 1 hour of operation.

2. Phenol degradation process

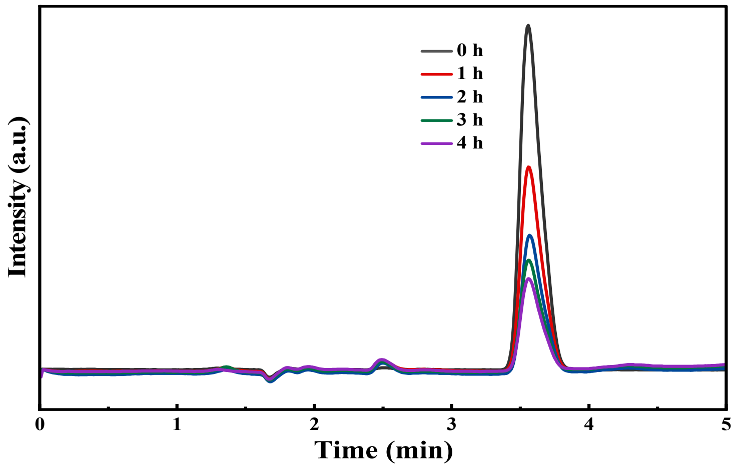

Figure S2. HPLC map of initial 5 ppm phenol to the map of phenol after 4 h of photocatalytic degradation.

The degradation process of phenol and the formation of intermediate products were investigated using high-performance liquid chromatography (HPLC) (Figure S2). The peak at 3.557 min corresponds to phenol; after 4 hours of light exposure, the intensity of this peak gradually decreased due to photocatalytic reactions. No background peaks were observed in the chromatogram, indicating complete mineralization of phenol.

3. Effect of catalysts on the degradation of RhB after adding different scavengers

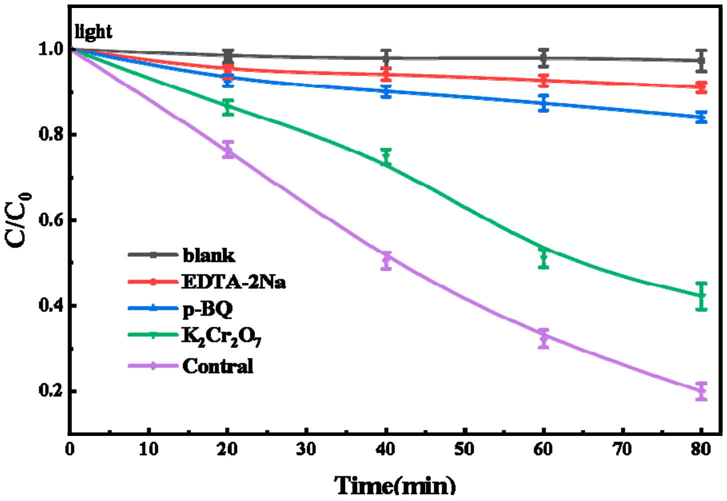

Figure S3. Effect of catalysts on the degradation of RhB after adding different scavengers [Reaction conditions: RhB = 10 mg·L-1 (50 mL), LED light source (λ > 420 nm), BP-5@TCNQ-0.3% dosage = 0.5 g·L-1, EDTA-2Na = 10 mM, p-BQ = 1 mM, $K_2Cr_2O_7$ = 0.5 mM, reaction time = 80 min, reaction temperature = 25◦C].
